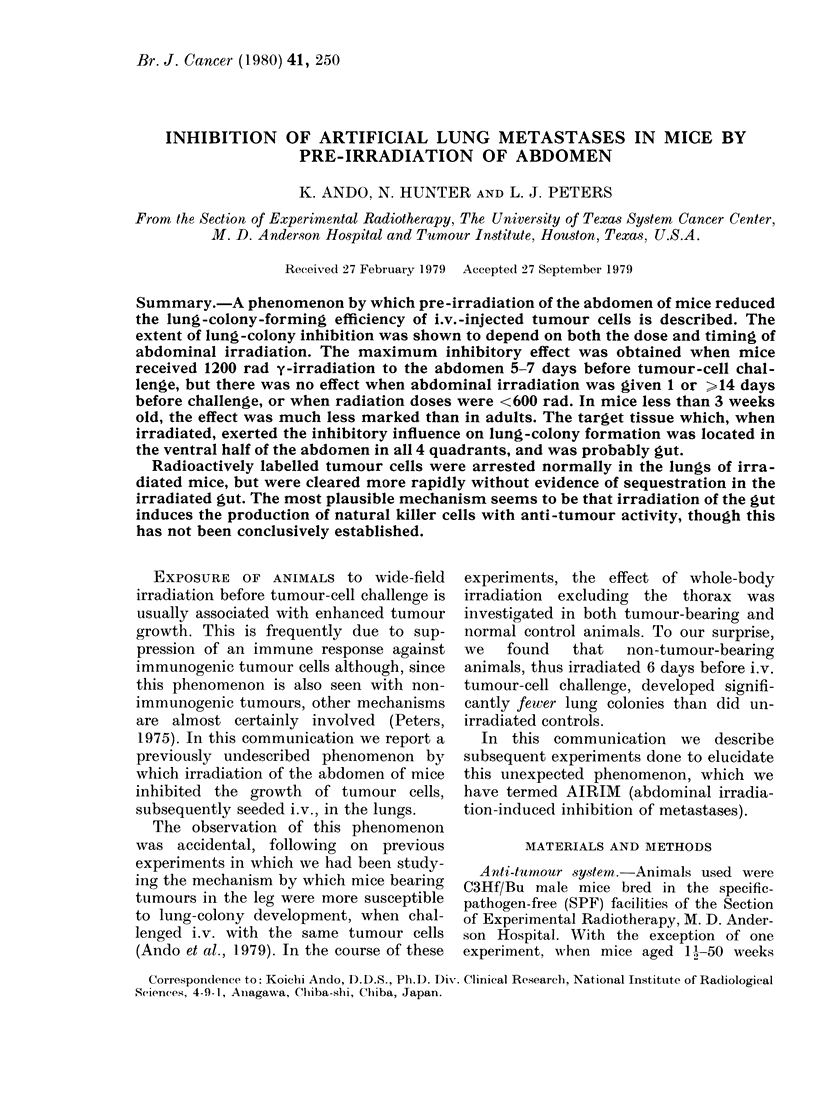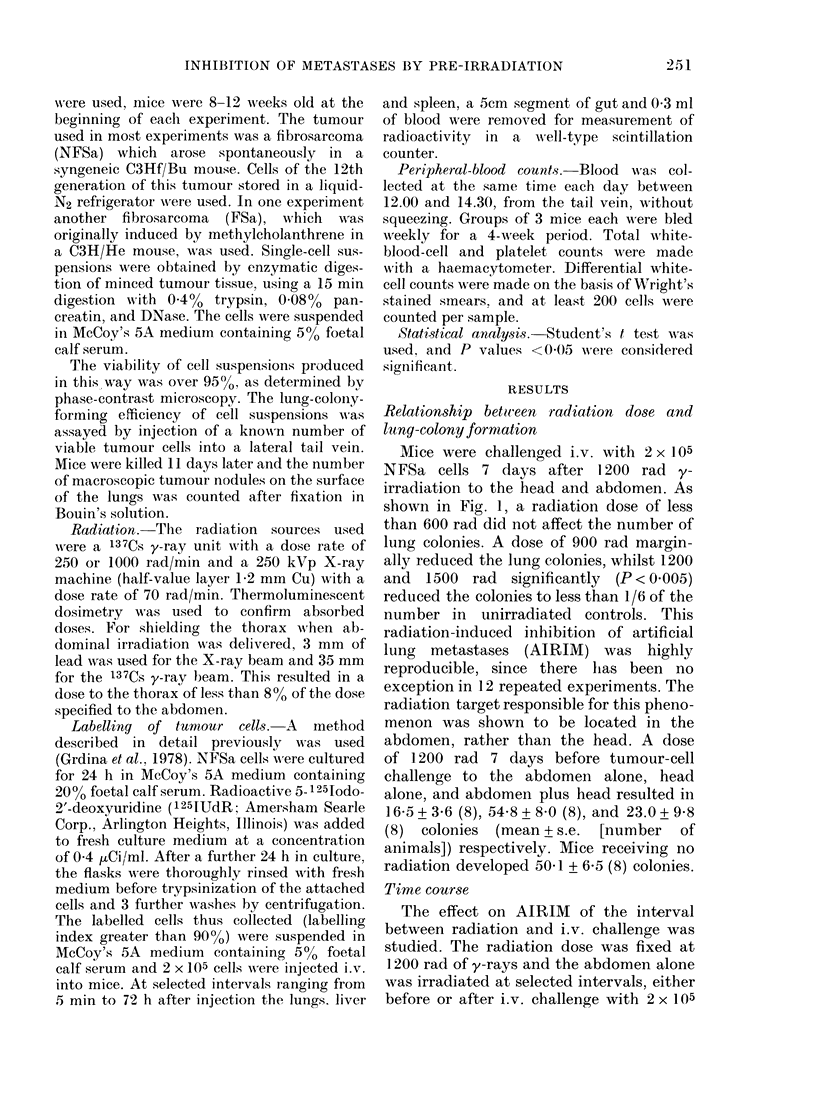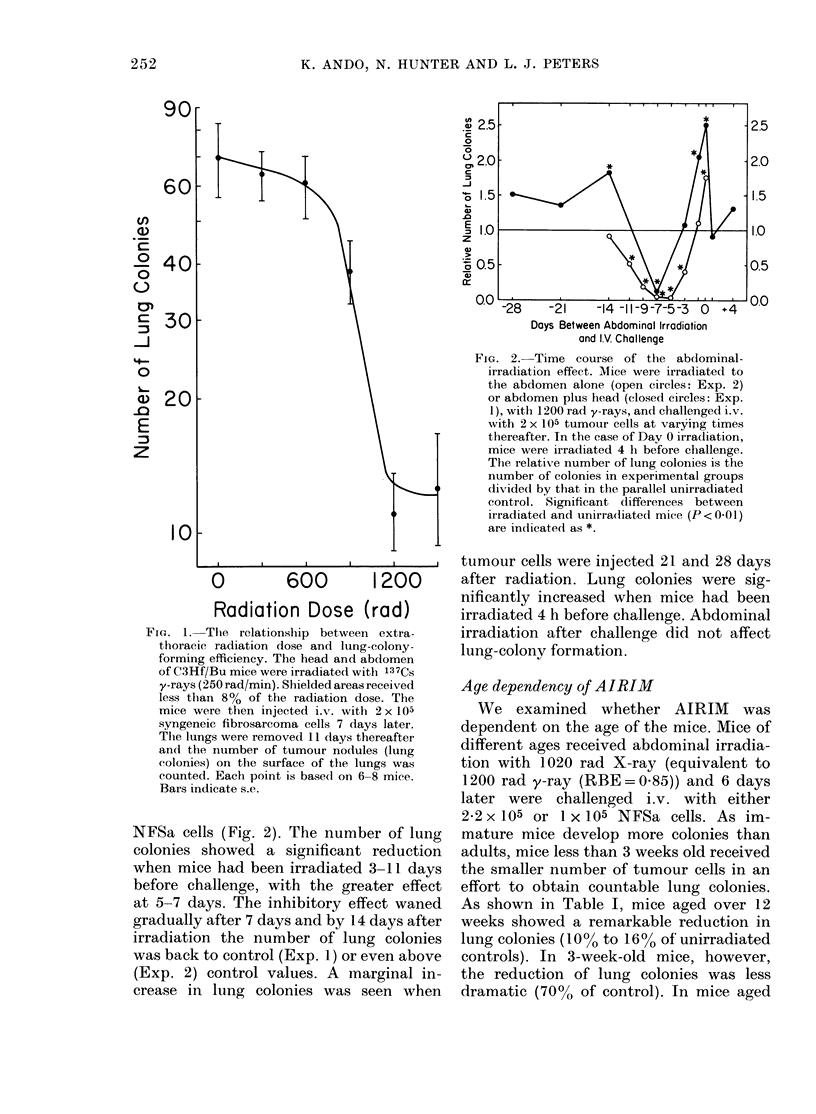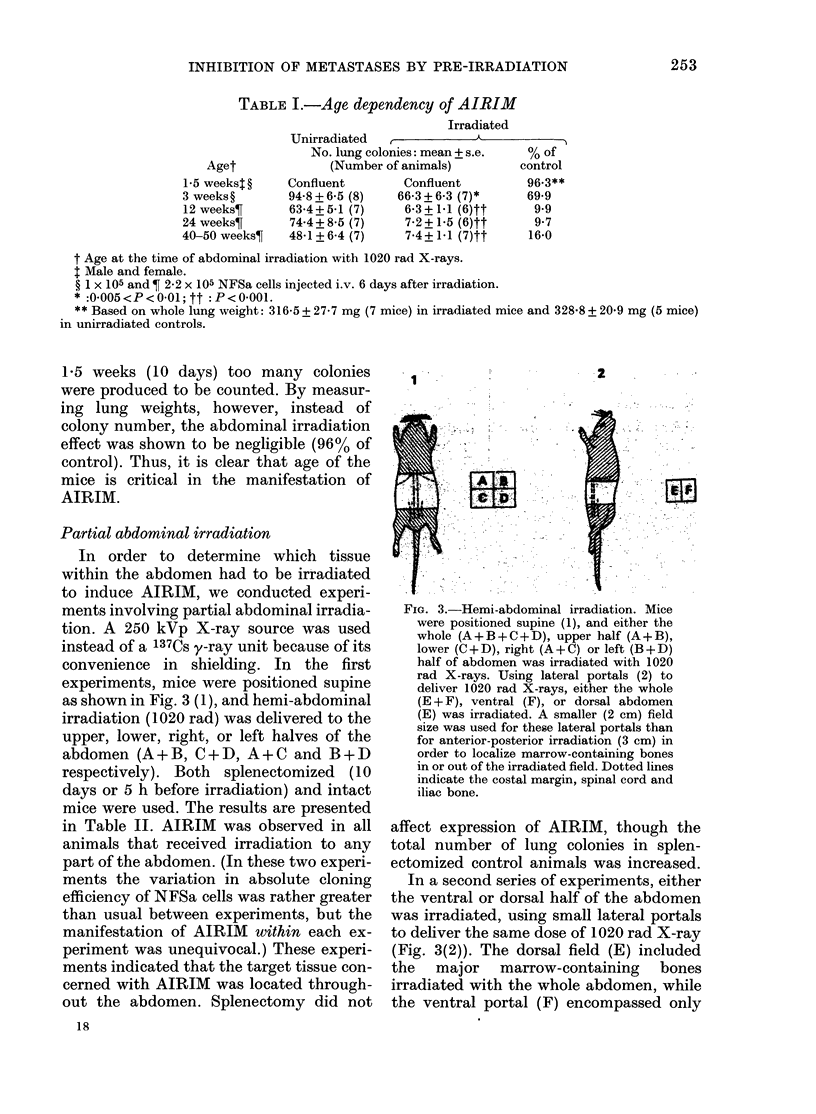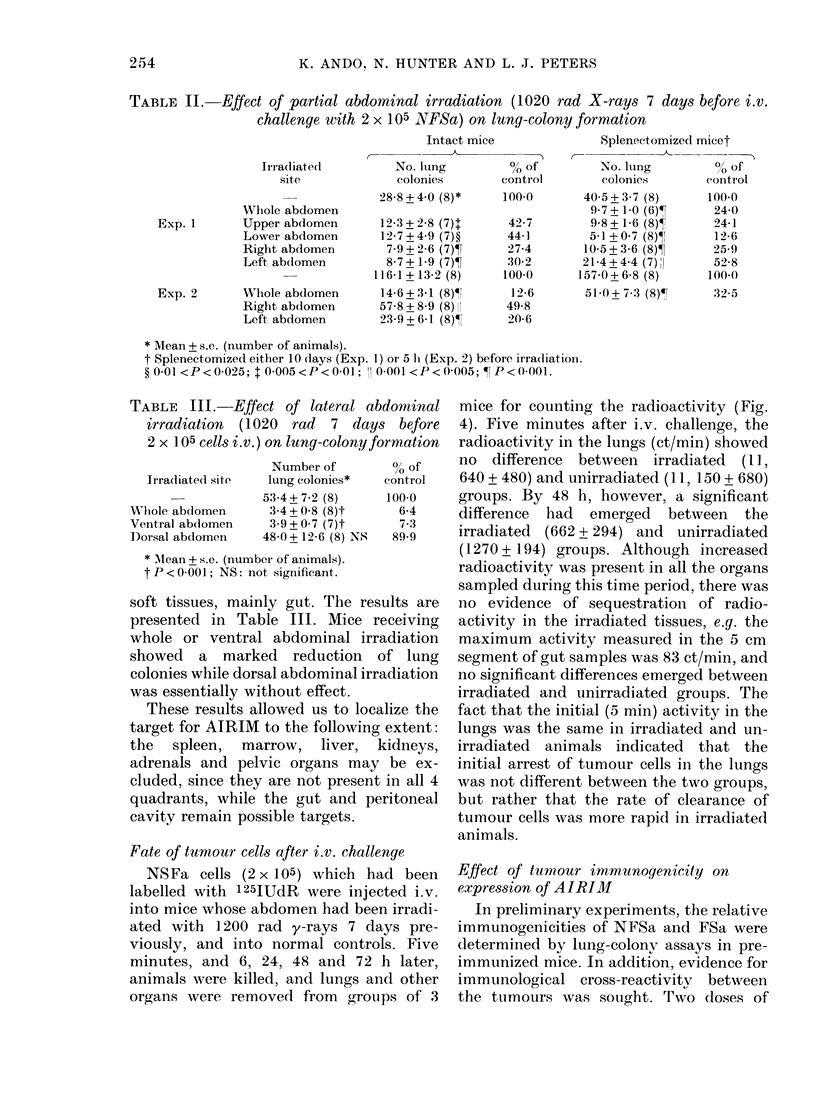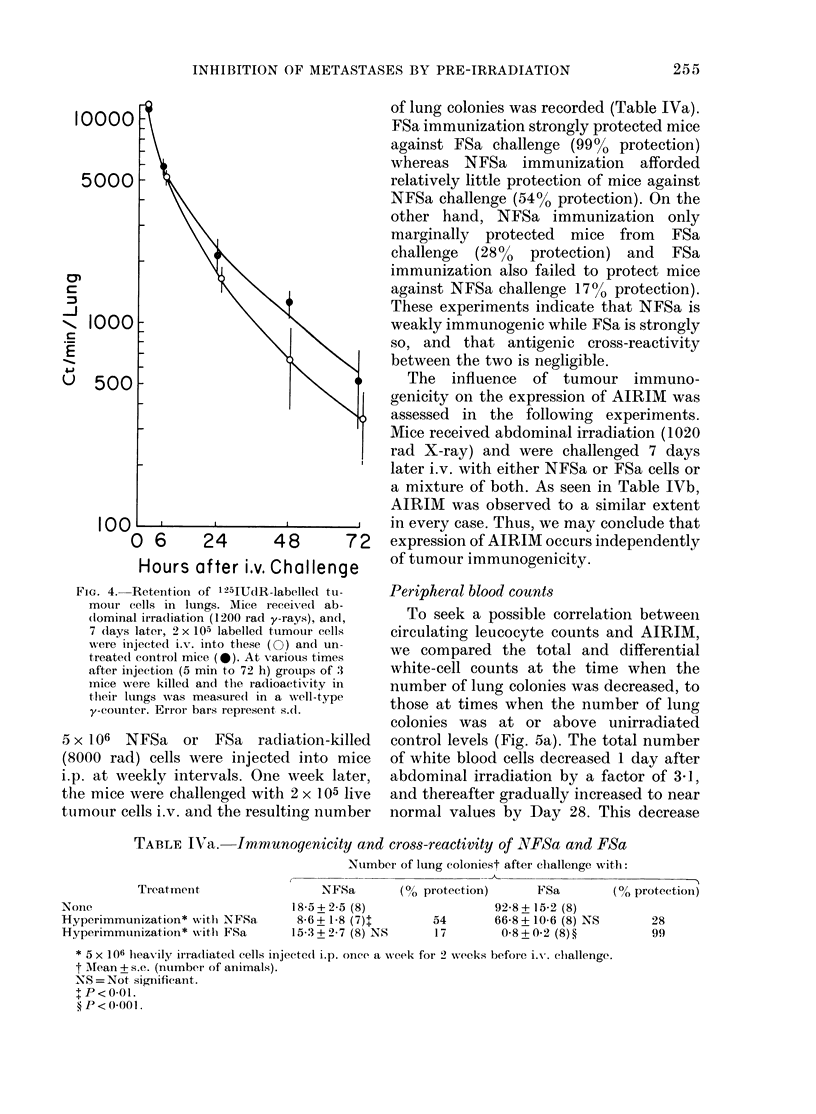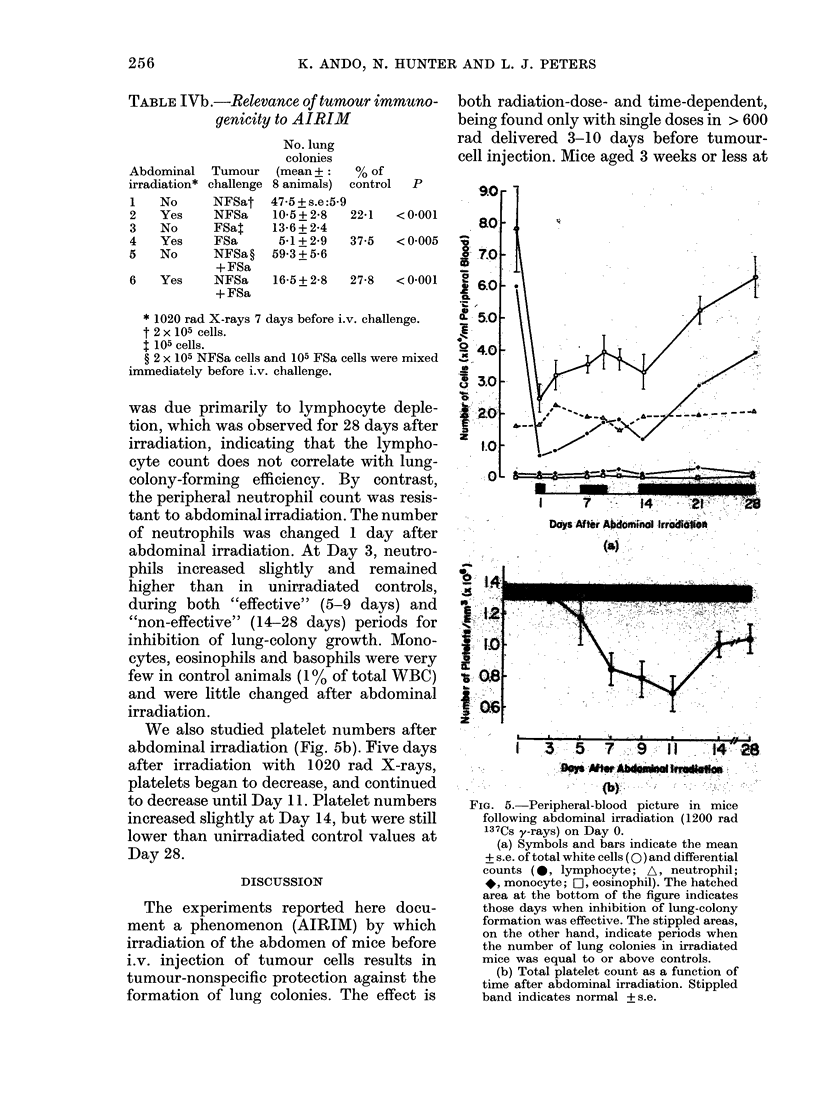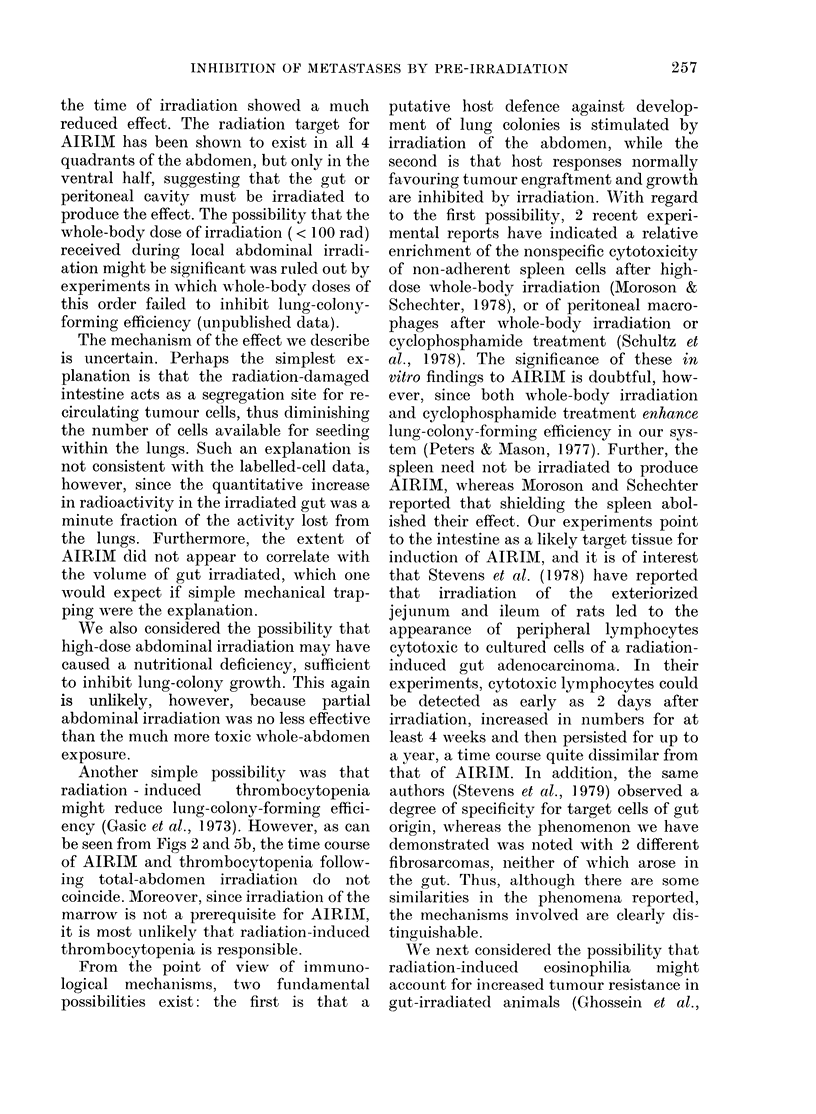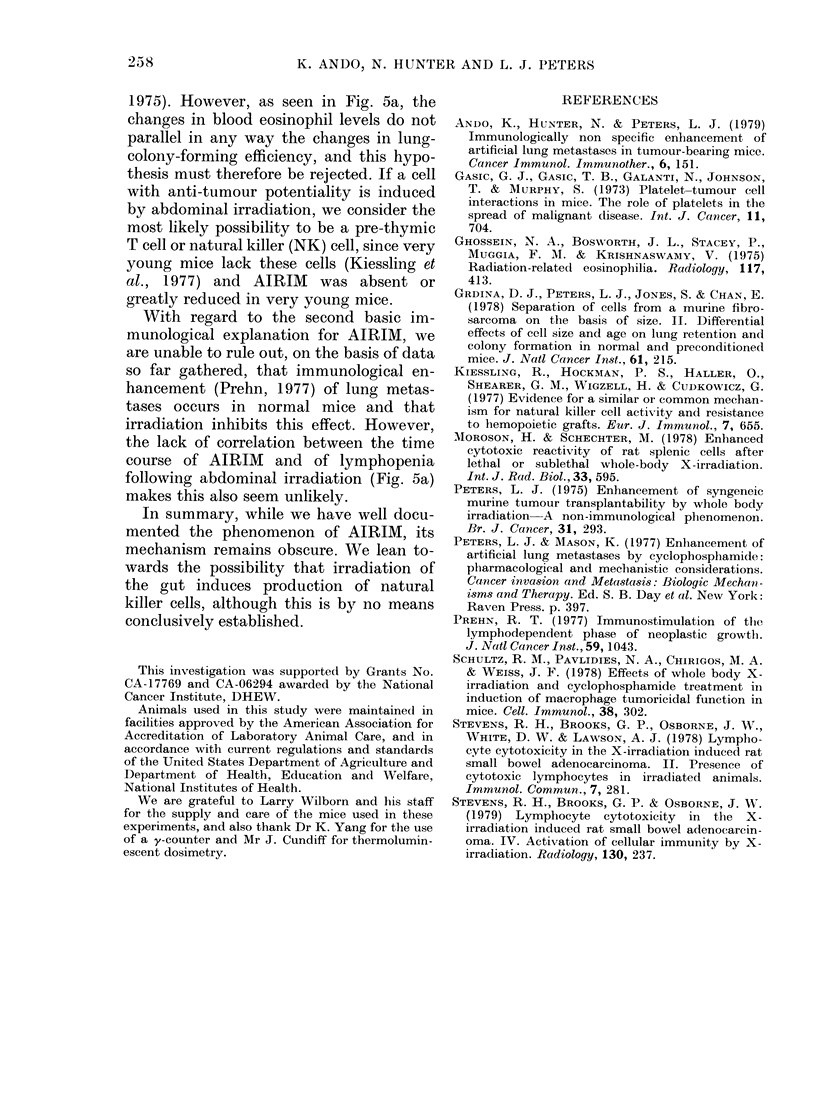# Inhibition of artificial lung metastases in mice by pre-irradiation of abdomen.

**DOI:** 10.1038/bjc.1980.37

**Published:** 1980-02

**Authors:** K. Ando, N. Hunter, L. J. Peters

## Abstract

A phenomenon by which pre-irradiation of the abdomen of mice reduced the lung-colony-forming efficiency of i.v.-injected tumour cells is described. The extent of lung-colony inhibition was shown to depend on both the dose and timing of abdominal irradiation. The maximum inhibitory effect was obtained when mice received 1200 rad gamma-irradiation to the abdomen 5--7 days before tumour-cell challenge, but there was no effect when abdominal irradiation was given 1 or greater than or equal to 14 days before challenge, or when radiation doses were less than 600 rad. In mice less than 3 weeks old, the effect was much less marked than in adults. The target tissue which, when irradiated, exerted the inhibitory influence on lung-colony formation was located in the ventral half of the abdomen in all 4 quadrants, and was probably gut. Radioactively labelled tumour cells were arrested normally in the lungs of irradiated mice, but were cleared more rapidly without evidence of sequestration in the irradiated gut. The most plausible mechanism seems to be that irradiation of the gut induces the production of natrual killer cells with anti-tumour activity, though this has not been conclusively established.


Br. J. Cancer (1980) 41, 250

### INHIBITION OF ARTIFICIAL LUNG METASTASES IN MICE BY PRE-IRRADIATION OF ABDOMEN

K. ANDO, N. HUNTER AND L. J. PETERS

*From the Section of Experimental Radiotherapy, The University of Texas System Cancer Center, M. D. Anderson Hospital and Tumour Institute, Houston, Texas, U.S.A.*

Received 27 February 1979 Accepted 27 September 1979

**Summary.—A phenomenon by which pre-irradiation of the abdomen of mice reduced the lung-colony-forming efficiency of i.v.-injected tumour cells is described. The extent of lung-colony inhibition was shown to depend on both the dose and timing of abdominal irradiation. The maximum inhibitory effect was obtained when mice received 1200 rad $\gamma$-irradiation to the abdomen 5–7 days before tumour-cell challenge, but there was no effect when abdominal irradiation was given 1 or $\geqslant$14 days before challenge, or when radiation doses were $<$600 rad. In mice less than 3 weeks old, the effect was much less marked than in adults. The target tissue which, when irradiated, exerted the inhibitory influence on lung-colony formation was located in the ventral half of the abdomen in all 4 quadrants, and was probably gut.**

**Radioactively labelled tumour cells were arrested normally in the lungs of irradiated mice, but were cleared more rapidly without evidence of sequestration in the irradiated gut. The most plausible mechanism seems to be that irradiation of the gut induces the production of natural killer cells with anti-tumour activity, though this has not been conclusively established.**

Exposure of animals to wide-field irradiation before tumour-cell challenge is usually associated with enhanced tumour growth. This is frequently due to suppression of an immune response against immunogenic tumour cells although, since this phenomenon is also seen with non-immunogenic tumours, other mechanisms are almost certainly involved (Peters, 1975). In this communication we report a previously undescribed phenomenon by which irradiation of the abdomen of mice inhibited the growth of tumour cells, subsequently seeded i.v., in the lungs.

The observation of this phenomenon was accidental, following on previous experiments in which we had been studying the mechanism by which mice bearing tumours in the leg were more susceptible to lung-colony development, when challenged i.v. with the same tumour cells (Ando *et al.*, 1979). In the course of these experiments, the effect of whole-body irradiation excluding the thorax was investigated in both tumour-bearing and normal control animals. To our surprise, we found that non-tumour-bearing animals, thus irradiated 6 days before i.v. tumour-cell challenge, developed significantly *fewer* lung colonies than did unirradiated controls.

In this communication we describe subsequent experiments done to elucidate this unexpected phenomenon, which we have termed AIRIM (abdominal irradiation-induced inhibition of metastases).

### MATERIALS AND METHODS

*Anti-tumour system.*—Animals used were C3Hf/Bu male mice bred in the specific-pathogen-free (SPF) facilities of the Section of Experimental Radiotherapy, M. D. Anderson Hospital. With the exception of one experiment, when mice aged $1\frac{1}{2}$–50 weeks

Correspondence to: Koichi Ando, D.D.S., Ph.D. Div. Clinical Research, National Institute of Radiological Sciences, 4-9-1, Anagawa, Chiba-shi, Chiba, Japan.

were used, mice were 8–12 weeks old at the beginning of each experiment. The tumour used in most experiments was a fibrosarcoma (NFSa) which arose spontaneously in a syngeneic C3Hf/Bu mouse. Cells of the 12th generation of this tumour stored in a liquid-$N_2$ refrigerator were used. In one experiment another fibrosarcoma (FSa), which was originally induced by methylcholanthrene in a C3H/He mouse, was used. Single-cell suspensions were obtained by enzymatic digestion of minced tumour tissue, using a 15 min digestion with 0·4% trypsin, 0·08% pancreatin, and DNase. The cells were suspended in McCoy's 5A medium containing 5% foetal calf serum.

The viability of cell suspensions produced in this way was over 95%, as determined by phase-contrast microscopy. The lung-colony-forming efficiency of cell suspensions was assayed by injection of a known number of viable tumour cells into a lateral tail vein. Mice were killed 11 days later and the number of macroscopic tumour nodules on the surface of the lungs was counted after fixation in Bouin's solution.

*Radiation.*—The radiation sources used were a $^{137}Cs$ $\gamma$-ray unit with a dose rate of 250 or 1000 rad/min and a 250 kVp X-ray machine (half-value layer 1·2 mm Cu) with a dose rate of 70 rad/min. Thermoluminescent dosimetry was used to confirm absorbed doses. For shielding the thorax when abdominal irradiation was delivered, 3 mm of lead was used for the X-ray beam and 35 mm for the $^{137}Cs$ $\gamma$-ray beam. This resulted in a dose to the thorax of less than 8% of the dose specified to the abdomen.

*Labelling of tumour cells.*—A method described in detail previously was used (Grdina *et al.*, 1978). NFSa cells were cultured for 24 h in McCoy's 5A medium containing 20% foetal calf serum. Radioactive 5-$^{125}$Iodo-2′-deoxyuridine ($^{125}$IUdR; Amersham Searle Corp., Arlington Heights, Illinois) was added to fresh culture medium at a concentration of 0·4 $\mu$Ci/ml. After a further 24 h in culture, the flasks were thoroughly rinsed with fresh medium before trypsinization of the attached cells and 3 further washes by centrifugation. The labelled cells thus collected (labelling index greater than 90%) were suspended in McCoy's 5A medium containing 5% foetal calf serum and $2 \times 10^5$ cells were injected i.v. into mice. At selected intervals ranging from 5 min to 72 h after injection the lungs, liver and spleen, a 5cm segment of gut and 0·3 ml of blood were removed for measurement of radioactivity in a well-type scintillation counter.

*Peripheral-blood counts.*—Blood was collected at the same time each day between 12.00 and 14.30, from the tail vein, without squeezing. Groups of 3 mice each were bled weekly for a 4-week period. Total white-blood-cell and platelet counts were made with a haemacytometer. Differential white-cell counts were made on the basis of Wright's stained smears, and at least 200 cells were counted per sample.

*Statistical analysis.*—Student's *t* test was used, and *P* values $<0{\cdot}05$ were considered significant.

### RESULTS

### *Relationship between radiation dose and lung-colony formation*

Mice were challenged i.v. with $2 \times 10^5$ NFSa cells 7 days after 1200 rad $\gamma$-irradiation to the head and abdomen. As shown in Fig. 1, a radiation dose of less than 600 rad did not affect the number of lung colonies. A dose of 900 rad marginally reduced the lung colonies, whilst 1200 and 1500 rad significantly ($P < 0{\cdot}005$) reduced the colonies to less than 1/6 of the number in unirradiated controls. This radiation-induced inhibition of artificial lung metastases (AIRIM) was highly reproducible, since there has been no exception in 12 repeated experiments. The radiation target responsible for this phenomenon was shown to be located in the abdomen, rather than the head. A dose of 1200 rad 7 days before tumour-cell challenge to the abdomen alone, head alone, and abdomen plus head resulted in $16{\cdot}5 \pm 3{\cdot}6$ (8), $54{\cdot}8 \pm 8{\cdot}0$ (8), and $23.0 \pm 9{\cdot}8$ (8) colonies (mean ± s.e. [number of animals]) respectively. Mice receiving no radiation developed $50{\cdot}1 \pm 6{\cdot}5$ (8) colonies.

### *Time course*

The effect on AIRIM of the interval between radiation and i.v. challenge was studied. The radiation dose was fixed at 1200 rad of $\gamma$-rays and the abdomen alone was irradiated at selected intervals, either before or after i.v. challenge with $2 \times 10^5$

FIG. 1.—The relationship between extrathoracic radiation dose and lung-colony-forming efficiency. The head and abdomen of C3Hf/Bu mice were irradiated with $^{137}Cs$ $\gamma$-rays (250 rad/min). Shielded areas received less than 8% of the radiation dose. The mice were then injected i.v. with $2 \times 10^5$ syngeneic fibrosarcoma cells 7 days later. The lungs were removed 11 days thereafter and the number of tumour nodules (lung colonies) on the surface of the lungs was counted. Each point is based on 6–8 mice. Bars indicate s.e.

FIG. 2.—Time course of the abdominal-irradiation effect. Mice were irradiated to the abdomen alone (open circles: Exp. 2) or abdomen plus head (closed circles: Exp. 1), with 1200 rad $\gamma$-rays, and challenged i.v. with $2 \times 10^5$ tumour cells at varying times thereafter. In the case of Day 0 irradiation, mice were irradiated 4 h before challenge. The relative number of lung colonies is the number of colonies in experimental groups divided by that in the parallel unirradiated control. Significant differences between irradiated and unirradiated mice ($P < 0{\cdot}01$) are indicated as *.

NFSa cells (Fig. 2). The number of lung colonies showed a significant reduction when mice had been irradiated 3–11 days before challenge, with the greater effect at 5–7 days. The inhibitory effect waned gradually after 7 days and by 14 days after irradiation the number of lung colonies was back to control (Exp. 1) or even above (Exp. 2) control values. A marginal increase in lung colonies was seen when tumour cells were injected 21 and 28 days after radiation. Lung colonies were significantly increased when mice had been irradiated 4 h before challenge. Abdominal irradiation after challenge did not affect lung-colony formation.

*Age dependency of AIRIM*

We examined whether AIRIM was dependent on the age of the mice. Mice of different ages received abdominal irradiation with 1020 rad X-ray (equivalent to 1200 rad $\gamma$-ray (RBE $= 0{\cdot}85$)) and 6 days later were challenged i.v. with either $2{\cdot}2 \times 10^5$ or $1 \times 10^5$ NFSa cells. As immature mice develop more colonies than adults, mice less than 3 weeks old received the smaller number of tumour cells in an effort to obtain countable lung colonies. As shown in Table I, mice aged over 12 weeks showed a remarkable reduction in lung colonies (10% to 16% of unirradiated controls). In 3-week-old mice, however, the reduction of lung colonies was less dramatic (70% of control). In mice aged

TABLE I.—*Age dependency of AIRIM*

| Age† | Unirradiated No. lung colonies: mean ± s.e. (Number of animals) | Irradiated No. lung colonies: mean ± s.e. (Number of animals) | Irradiated % of control |
|---|---|---|---|
| 1·5 weeks‡ § | Confluent | Confluent | 96·3** |
| 3 weeks§ | 94·8 ± 6·5 (8) | 66·3 ± 6·3 (7)* | 69·9 |
| 12 weeks¶ | 63·4 ± 5·1 (7) | 6·3 ± 1·1 (6)†† | 9·9 |
| 24 weeks¶ | 74·4 ± 8·5 (7) | 7·2 ± 1·5 (6)†† | 9·7 |
| 40–50 weeks¶ | 48·1 ± 6·4 (7) | 7·4 ± 1·1 (7)†† | 16·0 |

† Age at the time of abdominal irradiation with 1020 rad X-rays.
‡ Male and female.
§ $1 \times 10^5$ and ¶ $2 \cdot 2 \times 10^5$ NFSa cells injected i.v. 6 days after irradiation.
* :$0 \cdot 005 < P < 0 \cdot 01$; †† : $P < 0 \cdot 001$.
** Based on whole lung weight: 316·5 ± 27·7 mg (7 mice) in irradiated mice and 328·8 ± 20·9 mg (5 mice) in unirradiated controls.

1·5 weeks (10 days) too many colonies were produced to be counted. By measuring lung weights, however, instead of colony number, the abdominal irradiation effect was shown to be negligible (96% of control). Thus, it is clear that age of the mice is critical in the manifestation of AIRIM.

*Partial abdominal irradiation*

In order to determine which tissue within the abdomen had to be irradiated to induce AIRIM, we conducted experiments involving partial abdominal irradiation. A 250 kVp X-ray source was used instead of a $^{137}Cs$ $\gamma$-ray unit because of its convenience in shielding. In the first experiments, mice were positioned supine as shown in Fig. 3 (1), and hemi-abdominal irradiation (1020 rad) was delivered to the upper, lower, right, or left halves of the abdomen (A + B, C + D, A + C and B + D respectively). Both splenectomized (10 days or 5 h before irradiation) and intact mice were used. The results are presented in Table II. AIRIM was observed in all animals that received irradiation to any part of the abdomen. (In these two experiments the variation in absolute cloning efficiency of NFSa cells was rather greater than usual between experiments, but the manifestation of AIRIM *within* each experiment was unequivocal.) These experiments indicated that the target tissue concerned with AIRIM was located throughout the abdomen. Splenectomy did not affect expression of AIRIM, though the total number of lung colonies in splenectomized control animals was increased.

FIG. 3.—Hemi-abdominal irradiation. Mice were positioned supine (1), and either the whole (A + B + C + D), upper half (A + B), lower (C + D), right (A + C) or left (B + D) half of abdomen was irradiated with 1020 rad X-rays. Using lateral portals (2) to deliver 1020 rad X-rays, either the whole (E + F), ventral (F), or dorsal abdomen (E) was irradiated. A smaller (2 cm) field size was used for these lateral portals than for anterior-posterior irradiation (3 cm) in order to localize marrow-containing bones in or out of the irradiated field. Dotted lines indicate the costal margin, spinal cord and iliac bone.

In a second series of experiments, either the ventral or dorsal half of the abdomen was irradiated, using small lateral portals to deliver the same dose of 1020 rad X-ray (Fig. 3(2)). The dorsal field (E) included the major marrow-containing bones irradiated with the whole abdomen, while the ventral portal (F) encompassed only

TABLE II.—*Effect of partial abdominal irradiation* (1020 *rad X-rays* 7 *days before i.v. challenge with* $2 \times 10^5$ *NFSa*) *on lung-colony formation*

| | Irradiated site | Intact mice: No. lung colonies | Intact mice: % of control | Splenectomized mice†: No. lung colonies | Splenectomized mice†: % of control |
|---|---|---|---|---|---|
| Exp. 1 | — | 28·8 ± 4·0 (8)* | 100·0 | 40·5 ± 3·7 (8) | 100·0 |
| | Whole abdomen | | | 9·7 ± 1·0 (6)¶ | 24·0 |
| | Upper abdomen | 12·3 ± 2·8 (7)‡ | 42·7 | 9·8 ± 1·6 (8)¶ | 24·1 |
| | Lower abdomen | 12·7 ± 4·9 (7)§ | 44·1 | 5·1 ± 0·7 (8)¶ | 12·6 |
| | Right abdomen | 7·9 ± 2·6 (7)¶ | 27·4 | 10·5 ± 3·6 (8)¶ | 25·9 |
| | Left abdomen | 8·7 ± 1·9 (7)¶ | 30·2 | 21·4 ± 4·4 (7)‖ | 52·8 |
| Exp. 2 | — | 116·1 ± 13·2 (8) | 100·0 | 157·0 ± 6·8 (8) | 100·0 |
| | Whole abdomen | 14·6 ± 3·1 (8)¶ | 12·6 | 51·0 ± 7·3 (8)¶ | 32·5 |
| | Right abdomen | 57·8 ± 8·9 (8)‖ | 49·8 | | |
| | Left abdomen | 23·9 ± 6·1 (8)¶ | 20·6 | | |

* Mean ± s.e. (number of animals).

† Splenectomized either 10 days (Exp. 1) or 5 h (Exp. 2) before irradiation.

§ $0{\cdot}01 < P < 0{\cdot}025$; ‡ $0{\cdot}005 < P < 0{\cdot}01$; ‖ $0{\cdot}001 < P < 0{\cdot}005$; ¶ $P < 0{\cdot}001$.

TABLE III.—*Effect of lateral abdominal irradiation* (1020 *rad* 7 *days before* $2 \times 10^5$ *cells i.v.*) *on lung-colony formation*

| Irradiated site | Number of lung colonies* | % of control |
|---|---|---|
| — | 53·4 ± 7·2 (8) | 100·0 |
| Whole abdomen | 3·4 ± 0·8 (8)† | 6·4 |
| Ventral abdomen | 3·9 ± 0·7 (7)† | 7·3 |
| Dorsal abdomen | 48·0 ± 12·6 (8) NS | 89·9 |

* Mean ± s.e. (number of animals).
† $P < 0{\cdot}001$; NS: not significant.

soft tissues, mainly gut. The results are presented in Table III. Mice receiving whole or ventral abdominal irradiation showed a marked reduction of lung colonies while dorsal abdominal irradiation was essentially without effect.

These results allowed us to localize the target for AIRIM to the following extent: the spleen, marrow, liver, kidneys, adrenals and pelvic organs may be excluded, since they are not present in all 4 quadrants, while the gut and peritoneal cavity remain possible targets.

*Fate of tumour cells after i.v. challenge*

NSFa cells ($2 \times 10^5$) which had been labelled with $^{125}$IUdR were injected i.v. into mice whose abdomen had been irradiated with 1200 rad $\gamma$-rays 7 days previously, and into normal controls. Five minutes, and 6, 24, 48 and 72 h later, animals were killed, and lungs and other organs were removed from groups of 3 mice for counting the radioactivity (Fig. 4). Five minutes after i.v. challenge, the radioactivity in the lungs (ct/min) showed no difference between irradiated (11, 640 ± 480) and unirradiated (11, 150 ± 680) groups. By 48 h, however, a significant difference had emerged between the irradiated (662 ± 294) and unirradiated (1270 ± 194) groups. Although increased radioactivity was present in all the organs sampled during this time period, there was no evidence of sequestration of radioactivity in the irradiated tissues, *e.g.* the maximum activity measured in the 5 cm segment of gut samples was 83 ct/min, and no significant differences emerged between irradiated and unirradiated groups. The fact that the initial (5 min) activity in the lungs was the same in irradiated and unirradiated animals indicated that the initial arrest of tumour cells in the lungs was not different between the two groups, but rather that the rate of clearance of tumour cells was more rapid in irradiated animals.

*Effect of tumour immunogenicity on expression of AIRIM*

In preliminary experiments, the relative immunogenicities of NFSa and FSa were determined by lung-colony assays in preimmunized mice. In addition, evidence for immunological cross-reactivity between the tumours was sought. Two doses of

FIG. 4.—Retention of $^{125}$IUdR-labelled tumour cells in lungs. Mice received abdominal irradiation (1200 rad γ-rays), and, 7 days later, $2 \times 10^5$ labelled tumour cells were injected i.v. into these (○) and untreated control mice (●). At various times after injection (5 min to 72 h) groups of 3 mice were killed and the radioactivity in their lungs was measured in a well-type γ-counter. Error bars represent s.d.

$5 \times 10^6$ NFSa or FSa radiation-killed (8000 rad) cells were injected into mice i.p. at weekly intervals. One week later, the mice were challenged with $2 \times 10^5$ live tumour cells i.v. and the resulting number of lung colonies was recorded (Table IVa). FSa immunization strongly protected mice against FSa challenge (99% protection) whereas NFSa immunization afforded relatively little protection of mice against NFSa challenge (54% protection). On the other hand, NFSa immunization only marginally protected mice from FSa challenge (28% protection) and FSa immunization also failed to protect mice against NFSa challenge 17% protection). These experiments indicate that NFSa is weakly immunogenic while FSa is strongly so, and that antigenic cross-reactivity between the two is negligible.

The influence of tumour immunogenicity on the expression of AIRIM was assessed in the following experiments. Mice received abdominal irradiation (1020 rad X-ray) and were challenged 7 days later i.v. with either NFSa or FSa cells or a mixture of both. As seen in Table IVb, AIRIM was observed to a similar extent in every case. Thus, we may conclude that expression of AIRIM occurs independently of tumour immunogenicity.

*Peripheral blood counts*

To seek a possible correlation between circulating leucocyte counts and AIRIM, we compared the total and differential white-cell counts at the time when the number of lung colonies was decreased, to those at times when the number of lung colonies was at or above unirradiated control levels (Fig. 5a). The total number of white blood cells decreased 1 day after abdominal irradiation by a factor of 3·1, and thereafter gradually increased to near normal values by Day 28. This decrease

TABLE IVa.—*Immunogenicity and cross-reactivity of NFSa and FSa*

| | Number of lung colonies† after challenge with: | | | |
|---|---|---|---|---|
| Treatment | NFSa | (% protection) | FSa | (% protection) |
| None | 18·5 ± 2·5 (8) | | 92·8 ± 15·2 (8) | |
| Hyperimmunization* with NFSa | 8·6 ± 1·8 (7)‡ | 54 | 66·8 ± 10·6 (8) NS | 28 |
| Hyperimmunization* with FSa | 15·3 ± 2·7 (8) NS | 17 | 0·8 ± 0·2 (8)§ | 99 |

* $5 \times 10^6$ heavily irradiated cells injected i.p. once a week for 2 weeks before i.v. challenge.

† Mean ± s.e. (number of animals).

NS = Not significant.

‡ $P < 0{\cdot}01$.

§ $P < 0{\cdot}001$.

TABLE IVb.—*Relevance of tumour immunogenicity to AIRIM*

| | Abdominal irradiation* | Tumour challenge | No. lung colonies (mean ± : 8 animals) | % of control | *P* |
|---|---|---|---|---|---|
| 1 | No | NFSa† | 47·5 ± s.e :5·9 | | |
| 2 | Yes | NFSa | 10·5 ± 2·8 | 22·1 | < 0·001 |
| 3 | No | FSa‡ | 13·6 ± 2·4 | | |
| 4 | Yes | FSa | 5·1 ± 2·9 | 37·5 | < 0·005 |
| 5 | No | NFSa§ + FSa | 59·3 ± 5·6 | | |
| 6 | Yes | NFSa + FSa | 16·5 ± 2·8 | 27·8 | < 0·001 |

* 1020 rad X-rays 7 days before i.v. challenge.
† $2 \times 10^5$ cells.
‡ $10^5$ cells.
§ $2 \times 10^5$ NFSa cells and $10^5$ FSa cells were mixed immediately before i.v. challenge.

was due primarily to lymphocyte depletion, which was observed for 28 days after irradiation, indicating that the lymphocyte count does not correlate with lung-colony-forming efficiency. By contrast, the peripheral neutrophil count was resistant to abdominal irradiation. The number of neutrophils was changed 1 day after abdominal irradiation. At Day 3, neutrophils increased slightly and remained higher than in unirradiated controls, during both "effective" (5–9 days) and "non-effective" (14–28 days) periods for inhibition of lung-colony growth. Monocytes, eosinophils and basophils were very few in control animals (1% of total WBC) and were little changed after abdominal irradiation.

We also studied platelet numbers after abdominal irradiation (Fig. 5b). Five days after irradiation with 1020 rad X-rays, platelets began to decrease, and continued to decrease until Day 11. Platelet numbers increased slightly at Day 14, but were still lower than unirradiated control values at Day 28.

### DISCUSSION

The experiments reported here document a phenomenon (AIRIM) by which irradiation of the abdomen of mice before i.v. injection of tumour cells results in tumour-nonspecific protection against the formation of lung colonies. The effect is both radiation-dose- and time-dependent, being found only with single doses in > 600 rad delivered 3–10 days before tumour-cell injection. Mice aged 3 weeks or less at

FIG. 5.—Peripheral-blood picture in mice following abdominal irradiation (1200 rad $^{137}Cs$ $\gamma$-rays) on Day 0.

(a) Symbols and bars indicate the mean ± s.e. of total white cells (○) and differential counts (●, lymphocyte; △, neutrophil; ◆, monocyte; □, eosinophil). The hatched area at the bottom of the figure indicates those days when inhibition of lung-colony formation was effective. The stippled areas, on the other hand, indicate periods when the number of lung colonies in irradiated mice was equal to or above controls.

(b) Total platelet count as a function of time after abdominal irradiation. Stippled band indicates normal ± s.e.

the time of irradiation showed a much reduced effect. The radiation target for AIRIM has been shown to exist in all 4 quadrants of the abdomen, but only in the ventral half, suggesting that the gut or peritoneal cavity must be irradiated to produce the effect. The possibility that the whole-body dose of irradiation ($<100$ rad) received during local abdominal irradiation might be significant was ruled out by experiments in which whole-body doses of this order failed to inhibit lung-colony-forming efficiency (unpublished data).

The mechanism of the effect we describe is uncertain. Perhaps the simplest explanation is that the radiation-damaged intestine acts as a segregation site for recirculating tumour cells, thus diminishing the number of cells available for seeding within the lungs. Such an explanation is not consistent with the labelled-cell data, however, since the quantitative increase in radioactivity in the irradiated gut was a minute fraction of the activity lost from the lungs. Furthermore, the extent of AIRIM did not appear to correlate with the volume of gut irradiated, which one would expect if simple mechanical trapping were the explanation.

We also considered the possibility that high-dose abdominal irradiation may have caused a nutritional deficiency, sufficient to inhibit lung-colony growth. This again is unlikely, however, because partial abdominal irradiation was no less effective than the much more toxic whole-abdomen exposure.

Another simple possibility was that radiation - induced thrombocytopenia might reduce lung-colony-forming efficiency (Gasic *et al.*, 1973). However, as can be seen from Figs 2 and 5b, the time course of AIRIM and thrombocytopenia following total-abdomen irradiation do not coincide. Moreover, since irradiation of the marrow is not a prerequisite for AIRIM, it is most unlikely that radiation-induced thrombocytopenia is responsible.

From the point of view of immunological mechanisms, two fundamental possibilities exist: the first is that a putative host defence against development of lung colonies is stimulated by irradiation of the abdomen, while the second is that host responses normally favouring tumour engraftment and growth are inhibited by irradiation. With regard to the first possibility, 2 recent experimental reports have indicated a relative enrichment of the nonspecific cytotoxicity of non-adherent spleen cells after high-dose whole-body irradiation (Moroson & Schechter, 1978), or of peritoneal macrophages after whole-body irradiation or cyclophosphamide treatment (Schultz *et al.*, 1978). The significance of these *in vitro* findings to AIRIM is doubtful, however, since both whole-body irradiation and cyclophosphamide treatment *enhance* lung-colony-forming efficiency in our system (Peters & Mason, 1977). Further, the spleen need not be irradiated to produce AIRIM, whereas Moroson and Schechter reported that shielding the spleen abolished their effect. Our experiments point to the intestine as a likely target tissue for induction of AIRIM, and it is of interest that Stevens *et al.* (1978) have reported that irradiation of the exteriorized jejunum and ileum of rats led to the appearance of peripheral lymphocytes cytotoxic to cultured cells of a radiation-induced gut adenocarcinoma. In their experiments, cytotoxic lymphocytes could be detected as early as 2 days after irradiation, increased in numbers for at least 4 weeks and then persisted for up to a year, a time course quite dissimilar from that of AIRIM. In addition, the same authors (Stevens *et al.*, 1979) observed a degree of specificity for target cells of gut origin, whereas the phenomenon we have demonstrated was noted with 2 different fibrosarcomas, neither of which arose in the gut. Thus, although there are some similarities in the phenomena reported, the mechanisms involved are clearly distinguishable.

We next considered the possibility that radiation-induced eosinophilia might account for increased tumour resistance in gut-irradiated animals (Ghossein *et al.*,

1975). However, as seen in Fig. 5a, the changes in blood eosinophil levels do not parallel in any way the changes in lung-colony-forming efficiency, and this hypothesis must therefore be rejected. If a cell with anti-tumour potentiality is induced by abdominal irradiation, we consider the most likely possibility to be a pre-thymic T cell or natural killer (NK) cell, since very young mice lack these cells (Kiessling *et al.*, 1977) and AIRIM was absent or greatly reduced in very young mice.

With regard to the second basic immunological explanation for AIRIM, we are unable to rule out, on the basis of data so far gathered, that immunological enhancement (Prehn, 1977) of lung metastases occurs in normal mice and that irradiation inhibits this effect. However, the lack of correlation between the time course of AIRIM and of lymphopenia following abdominal irradiation (Fig. 5a) makes this also seem unlikely.

In summary, while we have well documented the phenomenon of AIRIM, its mechanism remains obscure. We lean towards the possibility that irradiation of the gut induces production of natural killer cells, although this is by no means conclusively established.

This investigation was supported by Grants No. CA-17769 and CA-06294 awarded by the National Cancer Institute, DHEW.

Animals used in this study were maintained in facilities approved by the American Association for Accreditation of Laboratory Animal Care, and in accordance with current regulations and standards of the United States Department of Agriculture and Department of Health, Education and Welfare, National Institutes of Health.

We are grateful to Larry Wilborn and his staff for the supply and care of the mice used in these experiments, and also thank Dr K. Yang for the use of a $\gamma$-counter and Mr J. Cundiff for thermoluminescent dosimetry.

### REFERENCES

Ando, K., Hunter, N. & Peters, L. J. (1979) Immunologically non specific enhancement of artificial lung metastases in tumour-bearing mice. *Cancer Immunol. Immunother.*, **6**, 151.

Gasic, G. J., Gasic, T. B., Galanti, N., Johnson, T. & Murphy, S. (1973) Platelet–tumour cell interactions in mice. The role of platelets in the spread of malignant disease. *Int. J. Cancer*, **11**, 704.

Ghossein, N. A., Bosworth, J. L., Stacey, P., Muggia, F. M. & Krishnaswamy, V. (1975) Radiation-related eosinophilia. *Radiology*, **117**, 413.

Grdina, D. J., Peters, L. J., Jones, S. & Chan, E. (1978) Separation of cells from a murine fibrosarcoma on the basis of size. II. Differential effects of cell size and age on lung retention and colony formation in normal and preconditioned mice. *J. Natl Cancer Inst.*, **61**, 215.

Kiessling, R., Hockman, P. S., Haller, O., Shearer, G. M., Wigzell, H. & Cudkowicz, G. (1977) Evidence for a similar or common mechanism for natural killer cell activity and resistance to hemopoietic grafts. *Eur. J. Immunol.*, **7**, 655.

Moroson, H. & Schechter, M. (1978) Enhanced cytotoxic reactivity of rat splenic cells after lethal or sublethal whole-body X-irradiation. *Int. J. Rad. Biol.*, **33**, 595.

Peters, L. J. (1975) Enhancement of syngeneic murine tumour transplantability by whole body irradiation—A non-immunological phenomenon. *Br. J. Cancer*, **31**, 293.

Peters, L. J. & Mason, K. (1977) Enhancement of artificial lung metastases by cyclophosphamide: pharmacological and mechanistic considerations. *Cancer invasion and Metastasis: Biologic Mechanisms and Therapy.* Ed. S. B. Day *et al.* New York: Raven Press. p. 397.

Prehn, R. T. (1977) Immunostimulation of the lymphodependent phase of neoplastic growth. *J. Natl Cancer Inst.*, **59**, 1043.

Schultz, R. M., Pavlidies, N. A., Chirigos, M. A. & Weiss, J. F. (1978) Effects of whole body X-irradiation and cyclophosphamide treatment in induction of macrophage tumoricidal function in mice. *Cell. Immunol.*, **38**, 302.

Stevens, R. H., Brooks, G. P., Osborne, J. W., White, D. W. & Lawson, A. J. (1978) Lymphocyte cytotoxicity in the X-irradiation induced rat small bowel adenocarcinoma. II. Presence of cytotoxic lymphocytes in irradiated animals. *Immunol. Commun.*, **7**, 281.

Stevens, R. H., Brooks, G. P. & Osborne, J. W. (1979) Lymphocyte cytotoxicity in the X-irradiation induced rat small bowel adenocarcinoma. IV. Activation of cellular immunity by X-irradiation. *Radiology*, **130**, 237.